# Is there an association between time of admission and in-hospital mortality in patients with non-ST-elevation myocardial infarction? A meta-analysis

**DOI:** 10.1038/srep14409

**Published:** 2015-09-22

**Authors:** Xiantao Wang, Jie Yan, Qiang Su, Yuhan Sun, Huafeng Yang, Lang Li

**Affiliations:** 1 Department of Cardiology, the First Affiliated Hospital of Guangxi Medical University, Nanning 530021, China; 2 Department of Clinical Laboratory, the First Affiliated Hospital of Guangxi Medical University, Nanning 530021, China

## Abstract

We performed a systematic review to assess whether being admitted during off-hours with non-ST-segment-elevation myocardial infarction (NSTEMI) is associated with increased in-hospital mortality. Previous studies have demonstrated an inconsistent association between patient arrival time for NSTEMI and the subsequent clinical outcomes. All studies published up to November 10, 2014 on the association between time of admission and mortality among patients with NSTEMI were identified by searching the MEDLINE, COCHRANE, EMBASE, and PUBMED databases. The characteristics and outcome data of the studies included in the systematic review were extracted. Summary odds ratios (ORs) and standardized mean differences (SMDs) with 95% confidence intervals (CIs) were calculated using a random-effects model. Five cohort studies with a total of 129,548 patients met our inclusion criteria. The pooled analysis demonstrated that off-hours admission was not associated with increased in-hospital mortality (OR = 1.02 [95% CI (0.93–1.13)], *P* = 0.687). Furthermore, off-hours admission did not result in a longer door-to-balloon time (SMD = 0.37, [95%CI:−0.002 to 0.73], *P* = 0.051). The in-hospital mortality of patients admitted with NSTEMI during off-hours was similar to that of patients admitted during regular hours. Time of admission may not be a risk factor for increased in-hospital mortality.

A number of studies have shown that patients with acute myocardial infarction (AMI) presenting during off-hours (weekday nights, weekends, and holidays) have higher mortality compared to those presenting during regular hours and that patients with ST-segment-elevation myocardial infarction (STEMI) have longer door-to-balloon times^1^. However, among patients with NSTEMI, findings regarding outcomes for patients admitted during off-hours are conflicting. While several studies showed an increased risk of death for patients admitted during off-hours^2^,^3^, others did not^4^,^5^,^6^. We postulated that patients admitted during off-hours may be at an increased risk of death. Therefore, we performed a systematic review to evaluate the available evidence on the association between off-hours admission and in-hospital mortality for patients with NSTEMI.

## Methods

### Search strategy

We performed a systematic search the MEDLINE, COCHRANE, EMBASE, and PUBMED databases up to November 10, 2014 with no restriction on date or language. The following keywords were used: “NSTEMI”, “time”, “off-hours”, “weekend”, “admission”, and “presentation”. References of relevant reports and review articles were also reviewed manually.

### Inclusion criteria

Studies were eligible if they compared the clinical outcomes between patients with NSTEMI admitted during off-hours to those admitted during regular hours. Studies were excluded for the following reasons: (a) lack of data on mortality; (b) published in abstract form only; (e) inclusion of participants less than 18 years of age; and (d) not published in a peer-reviewed journal.

### Data Extraction and Quality Assessment

Using a standardized data extraction protocol, two reviewers (X. T. Wang and J. Yan) independently extracted and collected the data. Disagreements or uncertainties were resolved by discussion of the two reviewers or by consultation with a third author (L. Li). When necessary, we attempted to contact the original authors for additional information. The extracted study characteristics included the first author, publication year, number of enrolled patients, study type, country, study period, definition of off-hours, variables adjusted for, and outcomes. We also abstracted the adjusted relative risk estimates (odds ratios [ORs]) of mortality with the corresponding 95% confidence intervals (CIs). Quality assessment was performed using the Newcastle-Ottawa Scale (NOS)^7^, which consists of 3 domains: cohort selection, comparability, and outcome. The maximum NOS score for an observational study is 9 points (4 points for selection, 2 points for comparability, and 3 points for outcome).

### Statistical analysis

A meta-analysis of the summary statistics from individual trials was performed using Stata software, version 12.0 (Stata Corporation, College Station, TX) by means of a DerSimonian and Laird random effects model^8^. The strength of the association between off-hours admission and in-hospital mortality for patients with NSTEMI was assessed by OR with 95% CI. The standardized mean difference (SMD) with 95% CI was used to express the pooled effect on measurement data. For continuous variables presented as medians with interquartile ranges (IQRs), the mean and standard deviation (SD) were estimated using the median and the estimator SD = IQR/1.35^9^. The significance of the pooled OR and SMD was determined by the Z test. Heterogeneity among studies was evaluated by a chi-square-based Q-test^10^. We considered a *P*_*Q*_ value < 0.1 as indicative of heterogeneity. For all other analyses, a *P* value < 0.05 was considered statistically significant. We performed subgroup analysis according to the definition of off-hours to explore the sources of heterogeneity among studies. Sensitivity analysis was performed by sequential omission of individual studies to assess the robustness of the results. Publication bias was evaluated by performing a linear regression of the standardized effect estimates against their precision according to the Egger’s test^11^.

## Results

### Search results

**Figure 1:**
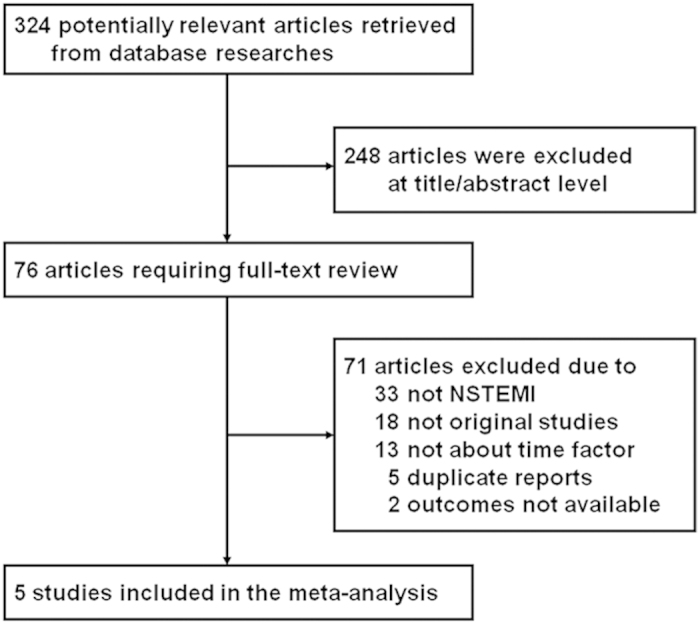
Flow chart for selection of eligible studies.

Our search strategy retrieved 324 potentially relevant articles, 76 of which were reviewed as full articles. Five studies that met our inclusion criteria were included in our systematic review and meta-analysis. Figure 1 summarizes the process of identifying eligible studies^2^,^3^,^4^,^5^,^6^.

### Study and patient characteristics

The characteristics of the selected studies are shown in Table 1, including author, publication year, number of enrolled patients, study type, country, study period, definition of off-hours, variables adjusted for, outcome assessed, and NOS score. All included studies had a cohort design. Four studies^2^,^3^,^4^,^6^ were conducted in North America (United States and Canada), and one study^5^ was conducted in Asia. The total pooled study population was 129,548 patients. All included studies had reported multivariate adjusted ORs for in-hospital mortality for patients admitted during off-hours and those admitted during regular hours. In three studies, the definition of off-hours included weekday nights^4^,^5^,^6^, while in the other two studies, the definition of off-hours included only weekends and holidays^2^,^3^.

### In-hospital mortality

**Figure 2:**
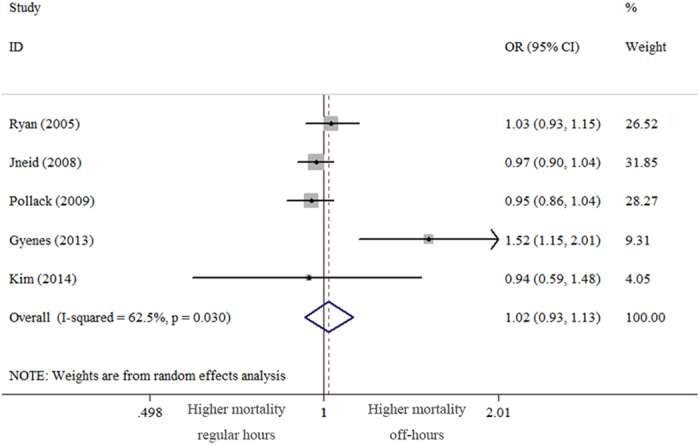
Forest plot of adjusted odds ratio for in-hospital mortality due to NSTEMI admitted during off-hours versus regular hours (random effects model with 95% CI).

In-hospital mortality was reported in all 5 studies, providing data for 129,548 patients with NSTEMI^2^,^3^,^4^,^5^,^6^. There was no difference in the adjusted odds of death between patients admitted during off-hours and those admitted during regular hours (OR = 1.02 [95% CI (0.93–1.13)], *P* = 0.687) (Fig. 2). Significant heterogeneity was observed among the studies (*P*_*Q*_ = 0.03). To explore the sources of heterogeneity, we performed subgroup analysis according to the definition of off-hours, with the following OR for death in patients admitted during off-hours: no weekday nights (OR = 0.96 [95% CI (0.91–1.02)], *P* = 0.187), heterogeneity *P*_*Q*_ = 0.938; and weekday nights (OR = 1.22 [95% CI (0.84–1.79)], *P* = 0.296), heterogeneity *P*_*Q*_ = 0.011.

### Door-to-balloon time

**Figure 3:**
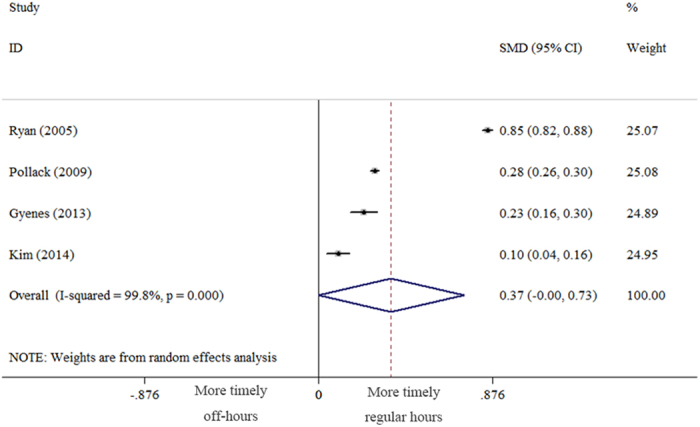
Forest plot of door-to-balloon time of NSTEMI patients admitted during off-hours versus regular hours (random effects model with 95% CI). SMD = standardized mean difference.

A total of 4 studies reported door-to-balloon time and provided data for 84,007 participants. The door-to-balloon time in patients with NSTEMI who were admitted during off-hours was not longer than that for those who were admitted during regular hours (SMD = 0.37, [95% CI:−0.002 to 0.73], *P* = 0.051) (Fig. 3). There was significant heterogeneity among the studies (*P*_*Q*_ < 0.001). We performed subgroup analysis according to the definition of off-hours to explore the sources of heterogeneity. The results were as follows: no weekday nights (SMD = 0.54, [95% CI: −0.07 to 1.15], *P* = 0.082), heterogeneity *P*_*Q*_ < 0.001; and weekday nights (SMD = 0.19, [95% CI: 0.01 to 0.37], *P* = 0.036), heterogeneity *P*_*Q*_ < 0.001.

### Sensitivity analysis

**Figure 4:**
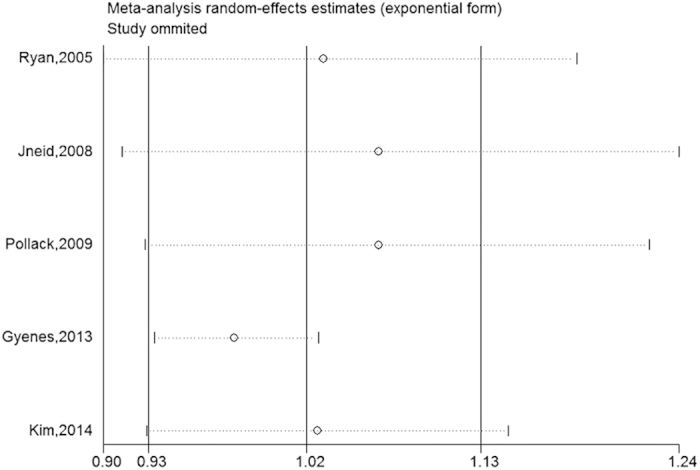
Results of the sensitivity analysis.

A sensitivity analysis was performed by removing one study at a time. The corresponding combined ORs were not materially altered after the removal of any study, suggesting that our results have high robustness (Fig. 4).

### Publication bias

**Figure 5:**
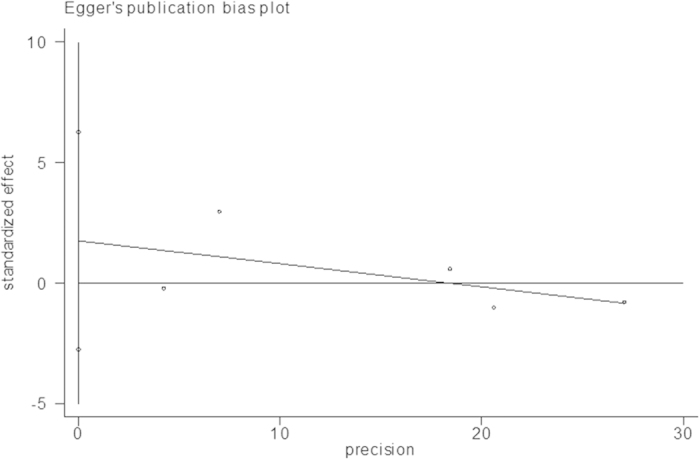
Egger’s plot assessing the publication bias of the included studies.

Linear regression of the standard normal deviate against precision suggested that the intercept did not significantly deviate from zero (*P* = 0.303). Therefore, according to Egger’s test, the results showed no evidence of publication bias (Fig. 5).

## Discussion

The major finding of this systematic review and meta-analysis is that in-hospital mortality of patients with NSTEMI admitted during off-hours was similar to those admitted during regular hours, even after adjusting for all patient covariates. Furthermore, off-hours admission did not result in longer door-to-balloon time; however, a subgroup analysis according to the definition of off-hours showed that weekday night admissions were associated with a longer door-to-balloon time. Off-hours admission may not be associated with a change in the risk of in-hospital mortality; however, significant heterogeneity was observed in the pooled analysis of individual study results.

According to our own survey of the literature, this is the first systematic review and meta-analysis to specifically assess the “off-hours effect” among NSTEMI patients. It is not entirely clear why admission during off-hours was not significantly associated with short-term mortality in this NSTEMI population.

Data regarding the “off-hours effect” in AMI are conflicting. Previous analyses revealed that patients with MI and heart failure who were admitted during off-hours had significantly higher adjusted in-hospital mortality^12^,^13^,^14^. These results were thought to be attributable to delayed reperfusion. Ting and colleagues found that admission during off-hours can increase the delay in reperfusion^15^. However, several other studies provided evidence that off-hours admission was not associated with in-hospital mortality in patients with MI^16^,^17^,^18^. The association between off-hours admission and mortality has also been evaluated in intensive care units (ICUs), and the results suggested that patients admitted to the ICU over the weekend have a higher risk of dying than those admitted on weekdays^19^. The lower level of staffing and intensity of care over the weekend may account for this finding^19^.

The importance of time to intervention to establish reperfusion is well-recognized in the STEMI population. In contrast, the optimal time to intervention has not clearly been elucidated in those with NSTEMI^20^. An early invasive strategy has not been shown to result in lower mortality in patients with NSTEMI. A related study suggested that the overall relationship between delay times and in-hospital mortality was generally not strong for patients with NSTEMI^21^. Our findings show that in patients with NSTEMI admitted during off-hours, door-to-balloon time may not be prolonged and in-hospital mortality may not increase.

A subgroup analysis of studies whose definition of off-hours included weekday nights showed that weekday night admissions were associated with a longer door-to-balloon time. Physicians have been shown to perform psychomotor tasks less proficiently at night^22^, and there are fewer health care professionals and experienced workers at night. We suspect that these factors might result in a longer door-to-balloon time. However, weekday night admissions were not associated with a change in in-hospital mortality. As long as a similar intensity of medical therapy is maintained, including the rates of antithrombotic therapy, antiplatelet therapy, and beta blocker use, possible delays in the timing of invasive treatment strategies for patients with NSTEMI do not result in a significantly increased death risk. However, a recent study found that off-hours admission was significantly associated with higher complication rates, especially ventricular arrhythmias and gastrointestinal bleeding^23^. Physicians should pay attention to this phenomenon and implement preventive measures. Regardless of the time of presentation, patients should have the same likelihood of receiving evidence-based treatment and timely reperfusion therapies, and hospitals should have the same number of hospital staff with the same level of expertise working at all times.

For better interpretation of the results, some limitations in this study should be acknowledged. First, the results were derived from observational studies; therefore, as with all observational data, residual confounding is possible. Second, the study population was not randomized. The patients’ baseline characteristics may confound the difference in mortality between patients admitted during off-hours and regular hours. Third, due to insufficient data, the following variables were not assessed: door-to-needle-time, composite major complications, and risk score. However, we believe that in-hospital mortality is one of the best tools to assess differences between the results of admission during regular hours and off-hours. Fourth, 5 studies were not enough to assess whether being admitted with NSTEMI during off-hours was associated with increased in-hospital mortality. The differences observed in this meta-analysis may have been caused by chance due to the small sample sizes of the included studies. Thus, the results regarding the relationship between off-hours admission and in-hospital mortality should be interpreted with caution. Finally, we were only able to compare in-hospital outcomes. It is unknown whether longer-term mortality is different between patients admitted during regular hours and those admitted during off-hours.

## Conclusions

In conclusion, our systematic review suggests that off-hours admission has limited impact on in-hospital mortality and door-to-balloon time among patients admitted with NSTEMI. Time of admission may not be a risk factor for increased in-hospital mortality.

## Additional Information

**How to cite this article**: Wang, X. *et al.* Is there an association between time of admission and in-hospital mortality in patients with non-ST-elevation myocardial infarction? A meta-analysis. *Sci. Rep.* **5**, 14409; doi: 10.1038/srep14409 (2015).

## Figures and Tables

**Table 1:** Characteristics of studies included in the meta-analysis.

First author, year	Country	Study period	Study type	Total No. of patients	Off-hours	Outcome assessed	Adjusted variables	NOS score
Ryan,2005	USA	2001–2003	Retrospective cohort	41,269	Between 5PM on Friday and 7AM on Sunday	In-hospital mortality, Door-to-balloon	Age, sex, race, hypertension, diabetes mellitus, smoking, hypercholesterolemia, prior CABG, positive cardiac markers	7
Jneid,2008	USA	2000–2005	Retrospective cohort	42,535	Weekends, holidays, and 7PM to 7AM weeknights	In-hospital mortality	Age, sex, race, BMI, insurance type, systolic BP, cardiac diagnosis, ST elevation or left bundle branch block, comorbidities	8
Pollack,2009	USA	2001–2003	Retrospective cohort	34,297	Weekends, holidays, and 7PM to 7AM weeknights	In-hospital mortality, Door-to-balloon	Age, sex, race, BMI, insurance status,smoking, family history of CAD,comorbidities, ischemic ST changes, signs of heart failure, heart rate, systolic BP, cardiologist care, hospital condition, teaching status, interventional capability	8
Gyenes,2013	Canada	1999–2008	Retrospective cohort	6,711	From Friday 4 PM until 4 PM of Sunday or 4 PM of the last day of the holiday	In-hospital mortality, Door-to-balloon	Age, Killip class, systolic BP, heart rate, initial creatinine, cardiac arrest at presentation, ST deviation, positive cardiac markers, history of TIA/stroke, on-site coronary angiography	9
Kim,2014	Korea	2005–2008	Prospective cohort	4,736	Weekdays 18:01 PM to 8:59AM, weekends, and holidays	In-hospital mortality, Door-to-balloon	age, gender, CPR, Killip class,primary VT, cardiovascular risk factors, previous MI, chronic heart failure, comorbidities, PCI performed,cardiogenic shock, left ventricular ejection fraction, GRACE risk score	9

BMI, body mass index; BP, blood pressure; CABG, coronary artery bypass grafting; CAD, coronary artery disease; CPR, cardiopulmonary resuscitation; GRACE, global registry of acute coronary events; MI, myocardial infarction; NOS, Newcastle-Ottawa Scale; TIA, transient ischemic attack; VT, ventricular tachyarrhythmia

## References

[b1] Sorita A. *et al.* Off-hour presentation and outcomes in patients with acute myocardial infarction: systematic review and meta-analysis. BMJ 348, f7393 (2014). 24452368 10.1136/bmj.f7393 PMC3898160

[b2] Ryan J. W. *et al.* Optimal timing of intervention in non-ST-segment elevation acute coronary syndromes: insights from the CRUSADE (Can Rapid risk stratification of Unstable angina patients Suppress ADverse outcomes with Early implementation of the ACC/AHA guidelines) Registry. Circulation 112, 3049–3057 (2005). 16275863 10.1161/CIRCULATIONAHA.105.582346

[b3] Gyenes G. T. *et al.* Use and timing of coronary angiography and associated in-hospital outcomes in Canadian non-ST-segment elevation myocardial infarction patients: insights from the Canadian Global Registry of Acute Coronary Events. Can. J. Cardiol. 29, 1429–1435 (2013). 23910228 10.1016/j.cjca.2013.04.035

[b4] Jneid H. *et al.* Impact of time of presentation on the care and outcomes of acute myocardial infarction. Circulation 117, 2502–2509 (2008). 18427127 10.1161/CIRCULATIONAHA.107.752113

[b5] Kim S. S. *et al.* Impact of patients’ arrival time on the care and in-hospital mortality in patients with non-ST-elevation myocardial infarction. Am. J. Cardiol. 113, 262–269 (2014). 24295548 10.1016/j.amjcard.2013.09.013

[b6] Pollack C. V. Jr. *et al.* Non-ST-elevation myocardial infarction patients who present during off hours have higher risk profiles and are treated less aggressively, but their outcomes are not worse: a report from Can Rapid Risk Stratification of Unstable Angina Patients Suppress ADverse Outcomes with Early Implementation of the ACC/AHA Guidelines CRUSADE initiative. Crit. Pathw. Cardiol . 8, 29–33 (2009). 19258835 10.1097/HPC.0b013e3181980f9f

[b7] Wells G. *et al.* *The Newcastle-Ottawa Scale (NOS) for assessing the quality of nonrandomised studies in meta-analyses*. Available at: http://www.ohri.ca/programs/clinical_epidemiology/oxford.asp. (Accessed: 10th November 2014).

[b8] DerSimonian R. & Laird N. Meta-analysis in clinical trials. Control. Clin. Trials 7, 177–188 (1986). 3802833 10.1016/0197-2456(86)90046-2

[b9] Higgins J. P. & Green S. *Cochrane handbook for systematic reviews of interventions, version 5.1.0. The Cochrane Collaboration*.(2011) Available at: http://www.cochrane-handbook.org. (Accessed: 18th March 2015).

[b10] Higgins J. P. & Thompson S. G. Quantifying heterogeneity in a meta-analysis. Stat. Med. 21, 1539–1558 (2002). 12111919 10.1002/sim.1186

[b11] Egger M., Davey Smith G., Schneider M. & Minder C. Bias in meta-analysis detected by a simple, graphical test. BMJ 315, 629–634 (1997). 9310563 10.1136/bmj.315.7109.629 PMC2127453

[b12] Magid D. J. *et al.* Relationship between time of day, day of week, timeliness of reperfusion, and in-hospital mortality for patients with acute ST-segment elevation myocardial infarction. JAMA 294, 803–812 (2005). 16106005 10.1001/jama.294.7.803

[b13] Kostis W. J. *et al.* Weekend versus weekday admission and mortality from myocardial infarction. N. Engl. J. Med. 356, 1099–1109 (2007). 17360988 10.1056/NEJMoa063355

[b14] Horwich T. B. *et al.* Weekend hospital admission and discharge for heart failure: association with quality of care and clinical outcomes. Am. Heart J. 158, 451–458 (2009). 19699870 10.1016/j.ahj.2009.06.025

[b15] Ting H. H. *et al.* Factors associated with longer time from symptom onset to hospital presentation for patients with ST-elevation myocardial infarction. Arch. Intern. Med. 168, 959–968 (2008). 18474760 10.1001/archinte.168.9.959 PMC4858313

[b16] Hong J. S., Kang H. C. & Lee S. H. Comparison of Case Fatality Rates for Acute Myocardial Infarction in Weekday vs Weekend Admissions in South Korea. Circulation Journal 74, 496–502 (2010). 20075558 10.1253/circj.cj-09-0678

[b17] Ortolani P. *et al.* Clinical comparison of “normal-hours” vs “off-hours” percutaneous coronary interventions for ST-elevation myocardial infarction. Am. Heart J. 154, 366–372 (2007). 17643590 10.1016/j.ahj.2007.04.025

[b18] Berger A. *et al.* Comparison of in-hospital mortality for acute myocardial infarction in Switzerland with admission during routine duty hours versus admission during out of hours (insight into the AMIS plus registry). Am. J. Cardiol. 101, 422–427 (2008). 18312751 10.1016/j.amjcard.2007.09.092

[b19] Cavallazzi R. *et al.* Association between time of admission to the ICU and mortality: a systematic review and metaanalysis. Chest 138, 68–75 (2010). 20418364 10.1378/chest.09-3018

[b20] Anderson J. L. *et al.* 2012 ACCF/AHA focused update incorporated into the ACCF/AHA 2007 guidelines for the management of patients with unstable angina/non-ST-elevation myocardial infarction: a report of the American College of Cardiology Foundation/American Heart Association Task Force on Practice Guidelines. J. Am. Coll. Cardiol. 61, e179–347 (2013). 23639841 10.1016/j.jacc.2013.01.014

[b21] Ting H. H. *et al.* Delay from symptom onset to hospital presentation for patients with non-ST-segment elevation myocardial infarction. Arch. Intern. Med. 170, 1834–1841 (2010). 21059977 10.1001/archinternmed.2010.385

[b22] Rollinson D. C. *et al.* The effects of consecutive night shifts on neuropsychological performance of interns in the emergency department: a pilot study. Ann. Emerg. Med. 41, 400–406 (2003). 12605209 10.1067/mem.2003.77

[b23] Sorita A. *et al.* Off-hour admission and outcomes for patients with acute myocardial infarction undergoing percutaneous coronary interventions. Am. Heart J. 169, 62–68 (2015). 25497249 10.1016/j.ahj.2014.08.012

